# Arthroscopic All-Suture Anchor Technique for Unstable Ramp Lesions with Medial Meniscal Defects

**DOI:** 10.1016/j.eats.2023.06.015

**Published:** 2023-09-25

**Authors:** Kentaro Miyamoto, Kazutoshi Kurokouchi, Shinya Ishizuka, Shigeo Takahashi, Masaru Idota, Takahiro Haga, Shiro Imagama

**Affiliations:** a Department of Orthopaedic Surgery and Arthroscopy Center, Juko Memorial Hospital, Nagoya, Japan; b Department of Orthopedic Surgery, Nagoya University Graduate School of Medicine, Nagoya, Japan

## Abstract

A ramp lesion is a specific type of tear in the meniscocapsular junction of the posterior horn of the medial meniscus, usually associated with anterior cruciate ligament (ACL) injury. Biomechanical cadaveric studies have shown that ACL injury combined with ramp lesions significantly increases anterior tibial translation and external rotation, which ACL reconstruction alone cannot completely control. Additionally, ramp lesions are sometimes associated with medial meniscal defects, especially in cases of chronic ACL deficiency after repetitive traumatic events, in which the anatomical repair of the meniscocapsular junction is infeasible. This report describes a new arthroscopic repair technique using an all-suture anchor through a posteromedial portal for unstable ramp lesions with medial meniscal defects.

A ramp lesion is a tear in the meniscocapsular junction of the posterior horn of the medial meniscus (PHMM) usually associated with anterior cruciate ligament (ACL) injury.^1^ The PHMM is firmly attached to the posterior edge of the tibia and serves as a secondary stabilizer to anterior tibial translation and external rotation.^2^ Biomechanical cadaveric studies have shown that ACL injury with ramp lesions significantly increases anterior tibial translation and external rotation, which ACL reconstruction alone cannot completely control.^3^ Missed ramp lesions during ACL reconstruction can lead to residual instability of the knee, graft failure, and secondary meniscus injury.^4^ Therefore ramp lesions should be repaired in conjunction with ACL reconstruction to restore optimal knee kinematics.

Studies have reported that unstable ramp lesions are usually fixed to the meniscocapsular complex and medial meniscus body using all-inside hooks from the posteromedial portal or disposable all-inside sutures from the anteromedial portal.^5^^,^^6^ Both methods have high fusion rates and good clinical outcomes. However, ramp lesions are sometimes associated with medial meniscal body defects, especially in cases of chronic ACL deficiency after repetitive traumatic events, in which case anatomic repair of the meniscocapsular junction is infeasible and knee joint instability remains.

This report describes a new arthroscopic repair technique using an all-suture anchor through a posteromedial portal for unstable ramp lesions with medial meniscal defects. Additionally, we report the good healing condition and stability of the lesion with second-look arthroscopy.

## Surgical Technique

### Preoperative Assessment

**Fig 1:**
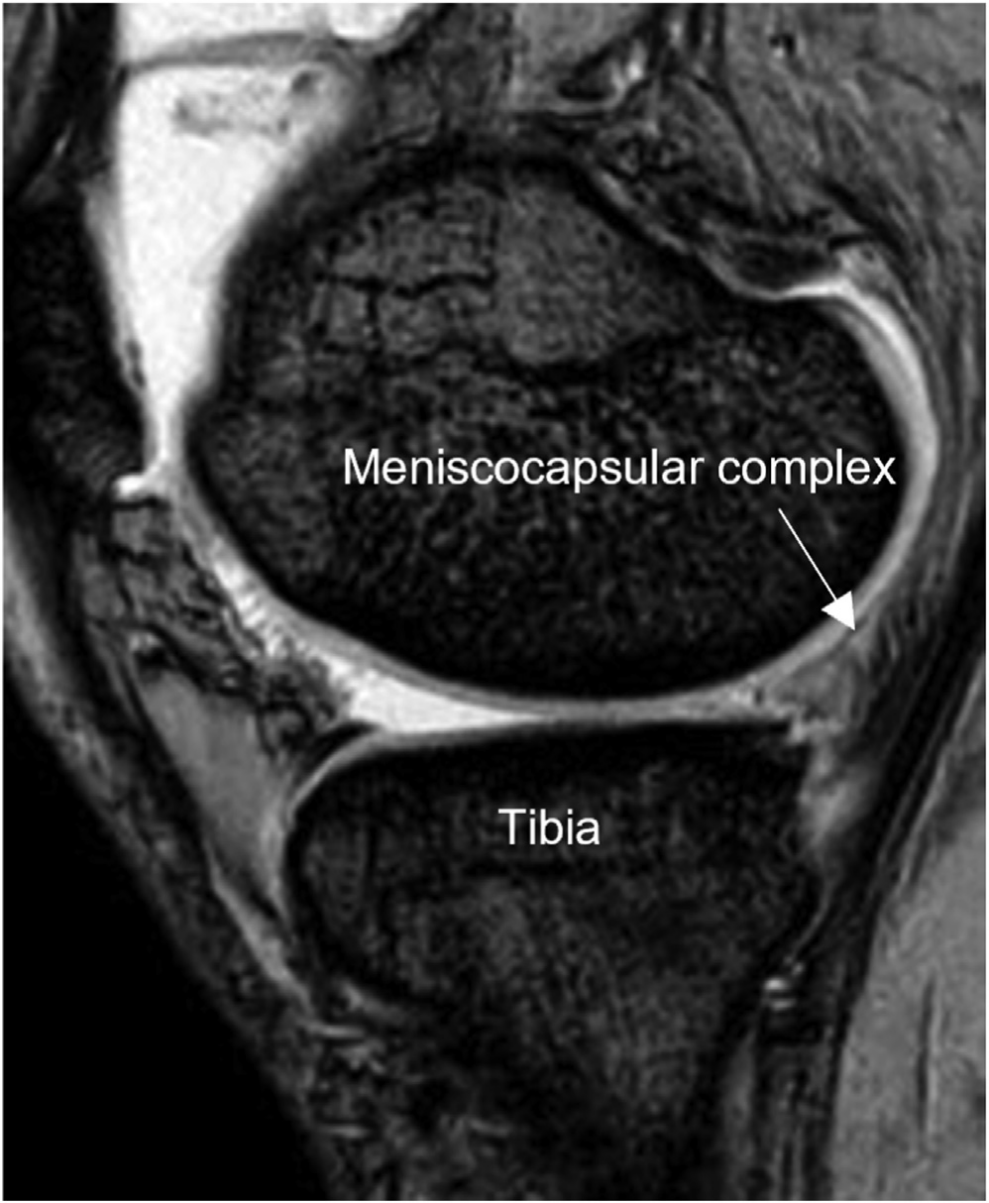
Sagittal magnetic resonance imaging of a left knee demonstrating posterior displacement of the meniscocapsular complex (white arrow) and anterior tibial subluxation.

The mechanism of injury, time from injury, and previous episodes of repetitive traumatic events are evaluated before surgery, followed by physical examination, including knee range of motion, pivot-shift test, and instrumented knee laxity measurement (KT-1000 arthrometer; Medmetric, San Diego, CA). All patients undergo magnetic resonance imaging of the knee to determine the ramp lesions complications and medial meniscal defects, and plan surgery. Figure 1 shows a typical ramp lesion magnetic resonance imaging scan bearing a medial meniscal defect.

### Arthroscopic Evaluation

The patient is positioned supine on the operating table, and a tourniquet is placed on the thigh. The knee is maintained at 90° of flexion using side and foot supports to enable stable performance of the arthroscopic procedure and to prevent popliteal and neural vascular lesions.^7^

**Fig 2:**
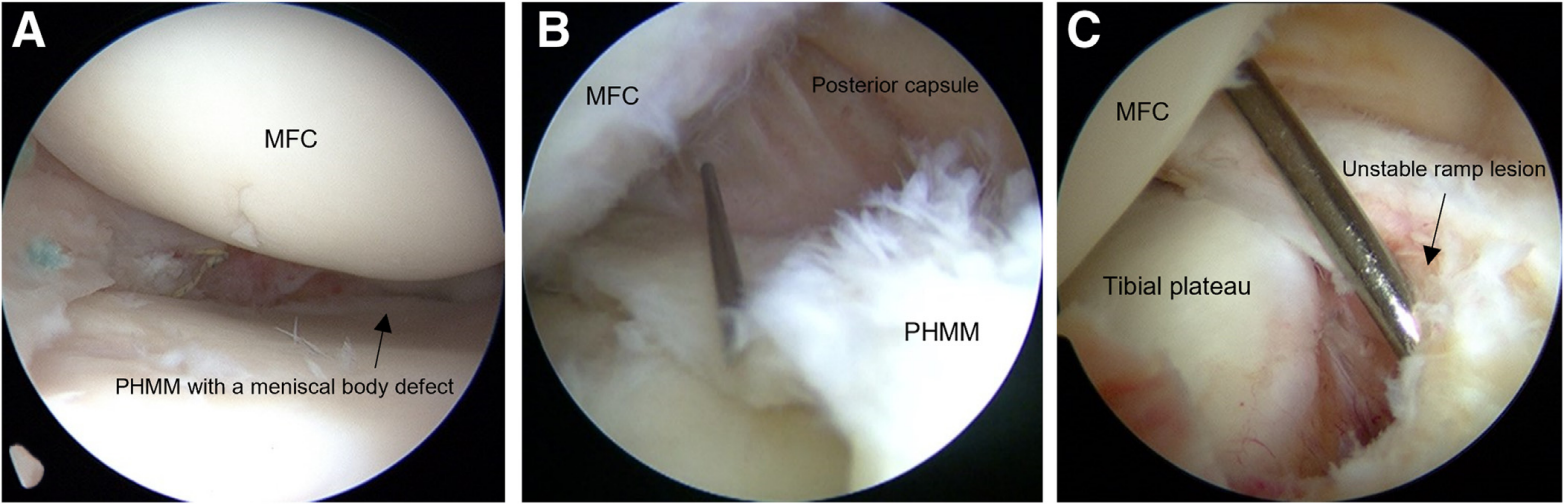
Visualization of the PHMM from a standard anterolateral portal. (A) The PHMM is associated with a meniscal body defect (black arrow). (B) The posteromedial portal is positioned using an outside-in technique with a 20-gauge spinal needle. (C) The unstable posteromedial meniscocapsular junction (black arrow) slides below the posterior margin of the tibia with knee flexion, thus exposing the tibial plateau. MFC, medial femoral condyle.

Diagnostic arthroscopy is performed using a standard anterolateral portal for the 45° arthroscope and anteromedial portal for the instruments (Video 1). The existence and pattern of meniscal tears are evaluated by carefully probing the meniscal tissues (Fig 2A). Ramp lesions are frequently overlooked by standard arthroscopy from only the anterior portals.^8^ The posteromedial compartment is assessed through the trans-notch view, which enables PHMM and posterior meniscocapsular junction visualization to confirm the presence of a ramp lesion.^9^

A 20-gauge spinal needle is inserted percutaneously into the posteromedial compartment of the knee and used as a probe to assess instability of the meniscocapsular junction (Fig 2 B and C). When the meniscocapsular junction, including the meniscocapsular and meniscotibial ligaments, is significantly unstable and detached from the posterior edge of the tibial plateau and the PHMM is associated with a meniscal body defect, the all-suture anchor technique from an additional posteromedial portal with an outside-in technique is used. In some cases, the unstable posteromedial meniscocapsular junction is slid below the tibial posterior margin at knee flexion to expose the tibial plateau.

### Suture Anchor Placement and Ramp Lesion Repair

**Fig 3:**
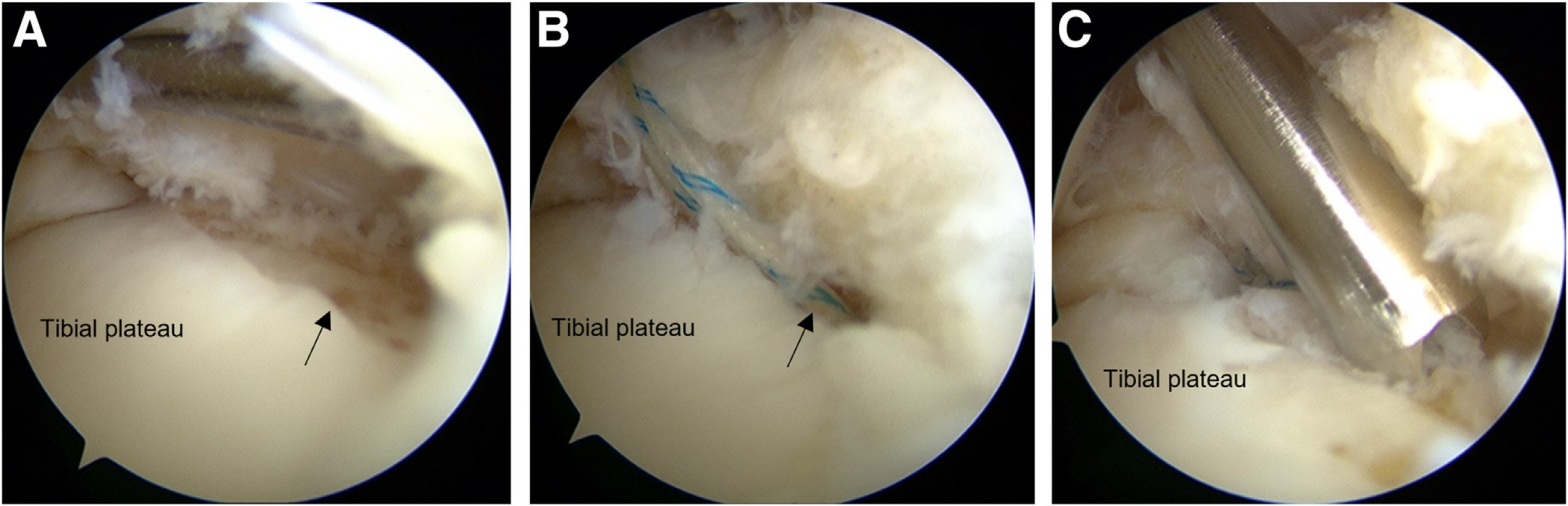
(A) The cartilage of the posterior margin of the tibia is abraded with a shaver (black arrow) to cause bleeding from the subchondral bone and to promote healing. (B) The all-suture anchors (black arrow) are placed 1 to 2 mm anterior to the posterior edge of the tibia. (C) A posteromedial portal using a curved anchor guide is used.

The tibial posterior margin cartilage is abraded with a shaver to create bleeding from the subchondral bone and promote healing (Fig 3A). Two or 3 all-suture anchors (Jugger Knot: Zimmer Biomet, Warsaw, IN; Q-Fix: Smith & Nephew, London, UK) are placed at the tibial margin through a posteromedial portal using a curved anchor guide (Fig 3 B and C). The first anchor is set on the medial side of the medial tibial plateau far from the posterior root of the medial meniscus and then toward the lateral side to avoid obstructing the arthroscopic view while working.

**Fig 4:**
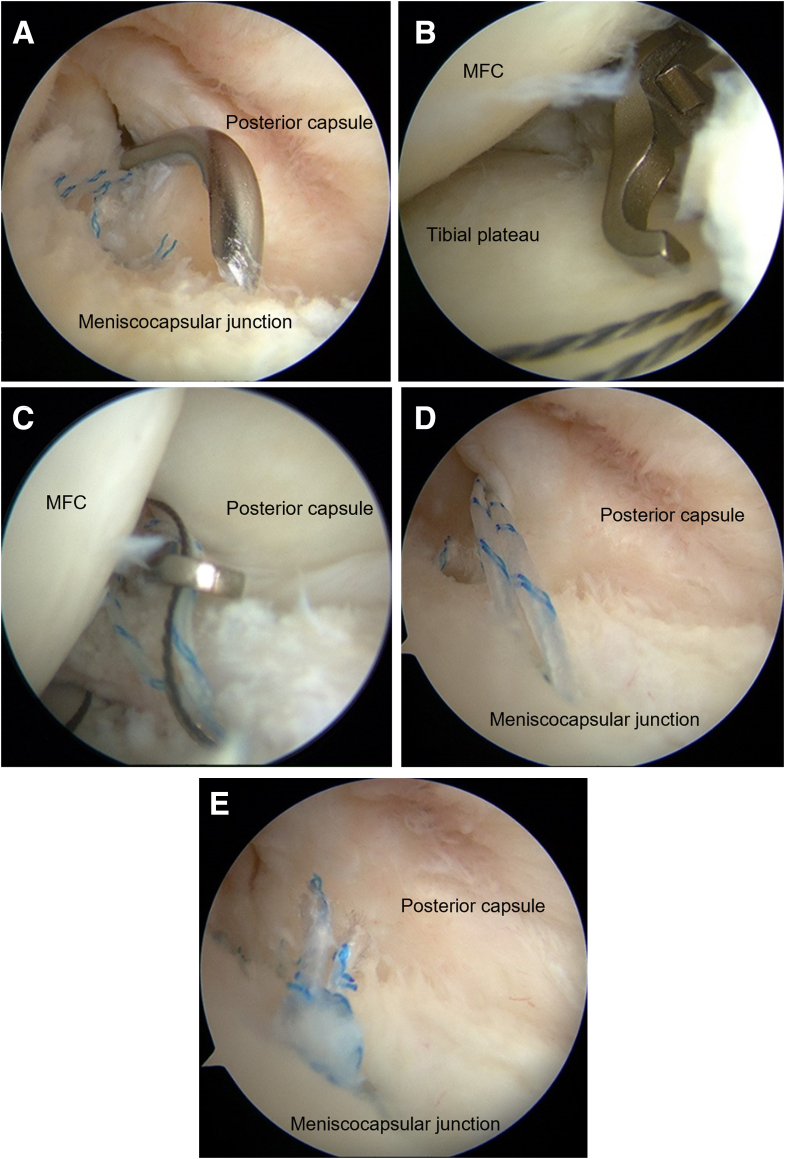
(A) The suture hook penetrates slightly posterior to the meniscocapsular junction to restore proper tension on the posterior capsule. (B) One of the suture tails and the wire of the suture hook are retrieved from the posteromedial portal. (C) The suture relay is carried out outside the joint. (D) The mattress sutures are tied with a sliding knot using a knot pusher. (E) The suture tails are cut using a suture cutter. MFC, medial femoral condyle.

A 90° angled suture hook device (Suture Lasso; Arthrex, Naples, FL) loaded with nitinol wire is inserted through the posteromedial portal and passed through the posterior capsule behind the meniscocapsular junction (Fig 4A). One of the suture tails and the wire of the suture hook are retrieved together from the posteromedial portal, and the suture relay is carried out outside the joint (Fig 4 B and C). The mattress sutures are tied with a sliding knot using a knot pusher, and the suture tails are cut with a suture cutter (Fig 4 D and E). This step is repeated for the remaining suture limbs of each anchor. A cannula is not preferred because it limits mobility; however, if the anchor sutures are difficult to retrieve or handle, a cannula is placed on the posteromedial portal. The anchors are placed 1 to 2 mm anterior to the posterior edge of the tibia, and the suture hook penetrates slightly posteriorly to the meniscocapsular junction to restore proper tension on the posterior capsule and increase the stability of the femorotibial joint. After the knots are tied, a probe is used to check the stability of the meniscocapsular junction and posteromedial capsule.

**Fig 5:**
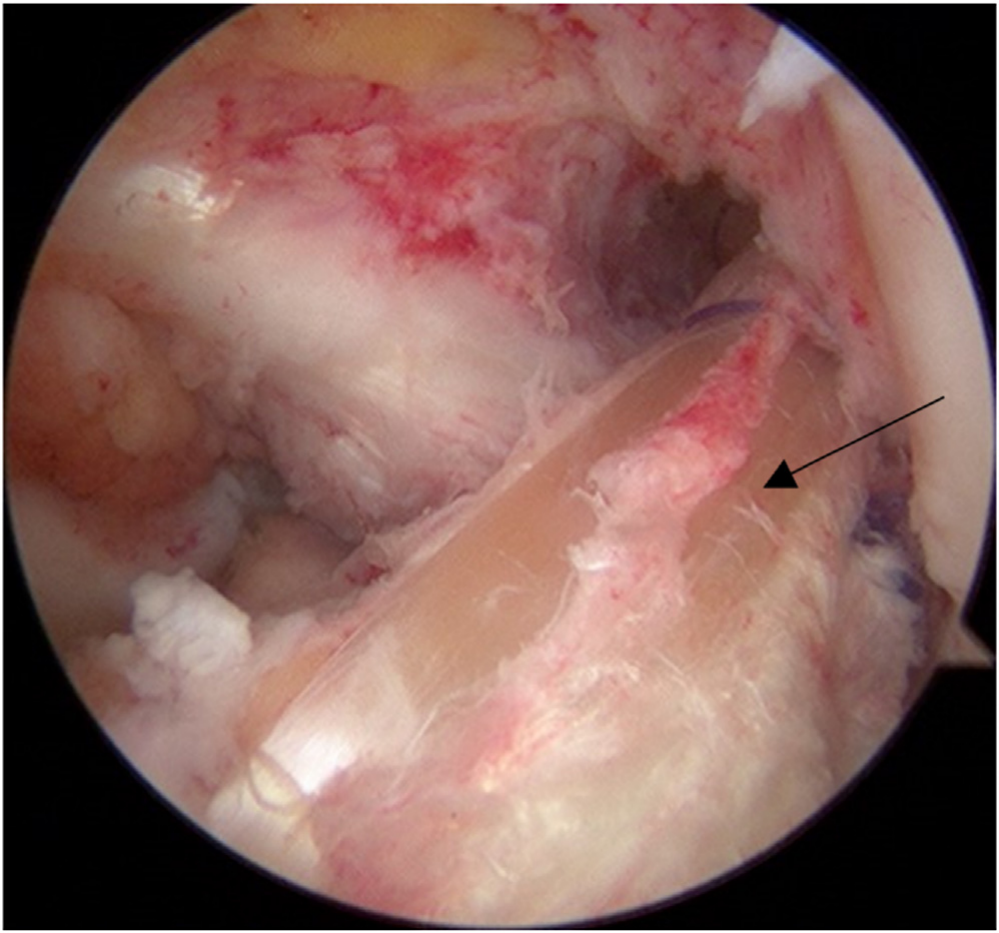
Anatomical double-bundle ACL reconstruction with a hamstring tendon autograft (black arrow) is performed using the outside-in tunnel technique.

Anatomical double-bundle ACL reconstruction with a hamstring tendon autograft is performed using the outside-in-tunnel technique (Fig 5). In cases of concomitant lateral meniscal tears, meniscal repair is performed in repairable cases, whereas partial meniscectomy is performed in irreparable cases.

### Postoperative Rehabilitation

The same postoperative rehabilitation protocol used for ACL reconstruction alone is followed. Active quadriceps exercises are performed, and passive motion exercises are started using a continuous passive motion machine on the second postoperative day. Two weeks after surgery, patients are allowed full weightbearing walking with a hinged brace that is worn for 3 months. Jogging is allowed at 3 months, with a gradual return to previous sports activities, including competitive sports, between 8 and 12 months after surgery.

### Second-Look Arthroscopy

**Fig 6:**
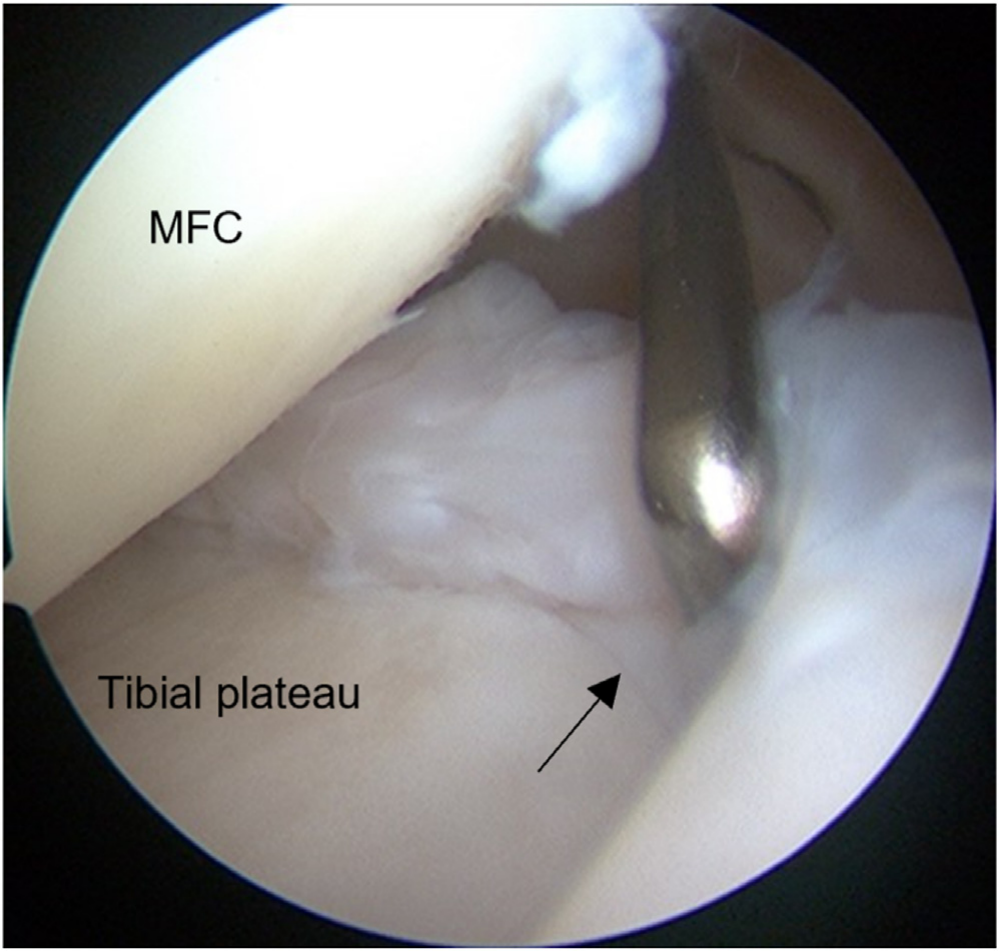
The meniscocapsular complex (black arrow) is firmly attached to the tibial plateau during the second-look arthroscopy.

Second-look arthroscopy and implant removal surgery for ACL reconstruction are performed between 12 and 24 months after surgery. The posteromedial compartment is assessed through the trans-notch view to probe the healing condition and stability of the meniscocapsular complex at the surface of the tibial plateau with an additional posteromedial portal. In all cases, in which second-look arthroscopy was possible, the meniscocapsular complex was firmly attached to the tibial plateau (Fig 6).

## Discussion

The medial meniscus functions as a shock absorber and load distribution and plays an important role in limiting anterior tibial translation against anterior tibial loading by acting as a “bumper” in ACL-deficient knees.^10^^,^^11^ Biomechanical studies have shown that ACL injury increases the tibial anterior translation and results in anterior tibial subluxation, causing the PHMM, which acts as a wedge against the posterior tibia, to engage the femoral condyle.^12^ Thereby, the PHMM meniscocapsular interface is subjected to high forces, which may lead to concomitant ramp lesions, defined as longitudinal tears of the PHMM meniscocapsular junction. This tear pattern has been described as the “Bankart” lesion of the knee.^11^

Unstable ramp lesions are usually sutured to the meniscocapsular complex and medial meniscus body, resulting in restoration of knee stability. However, ramp lesions are sometimes found with medial meniscus defects, especially in chronic cases or in cases of repeated episodes of giving way, in which case anatomic repair of the meniscocapsular junction is impossible, resulting in residual knee instability. Papageorgiou et al.^13^ demonstrated up to a 50% increase in the ACL replacement graft in situ forces after medial meniscectomy in response to a combined anterior and axial compressive tibial load.

The arthroscopic all-suture anchor technique shown here closely resembles the Bankart shoulder repair technique. In that case, the detached labrum is repaired to the glenoid using suture anchors to retension the joint capsule and restore glenohumeral joint stability. In this study, the meniscocapsular complex was repaired on the tibial posterior aspect using suture anchors to restore the posteromedial capsule stability. In addition, second-look arthroscopy showed good healing of the lesion.

The key point of this surgical technique is to use a curved anchor guide to insert the anchor through the posterior medial portal on the tibial posterior margin and use all-suture anchors with high pullout strength because the tibial posterior margin bone quality can be poor. In addition, a hook device is used to pass sutures through the meniscocapsular complex with sufficient thickness, and mattress sutures and tying with sliding knots can be used to obtain strong fixation. Pearls and pitfalls of our technique are summarized in Table 1. Although this technique presents technical difficulties in placing anchors and tying sutures, it is minimally invasive, requires few instruments, and improves knee stability, even in cases of medial meniscal defects. The advantages and disadvantages of our technique are summarized in Table 2.

**Table 1:** Pearls and Pitfalls of the Surgical Technique

Pearls
The posteromedial compartment is assessed through the trans-notch view using the 45° arthroscope.
The tibial posterior margin cartilage is abraded with a shaver to create bleeding from the subchondral bone and to promote healing.
Two or 3 all-suture anchors are placed at the tibial margin through a posteromedial portal using a curved anchor guide.
A 90° angled suture hook device is passed through the posterior capsule behind the meniscocapsular junction, and the mattress sutures are tied with a sliding knot.
Pitfalls
Ramp lesions are frequently overlooked by standard arthroscopy from only the anterior portals.
A cannula is not preferred, because it limits mobility.
All-suture anchors with high pullout strength are used because the tibial posterior margin bone quality can be poor.
A suture hook penetrates slightly posterior to the meniscocapsular junction to restore proper tension on the posterior capsule.

**Table 2:** Advantages and Disadvantages of the Surgical Technique

Advantages
It requires only 1 extra posteromedial portal.
It requires fewer instruments.
It improves knee stability even in the presence of medial meniscal defects.
It is easy to implement and results in good healing of the lesion.
Disadvantages
Placing anchors and tying sutures is technically difficult.
It requires additional surgical time.
It presents risk of saphenous nerve injury.

Taken together, we consider that treating unstable ramp lesions with medial meniscal defects using all-suture anchors through a posteromedial portal is an effective solution to restore knee joint stability and optimal knee kinematics in a minimally invasive procedure. A limitation includes the absence of currently available clinical outcome studies of this surgical technique; therefore further research is needed to investigate its clinical results.

## Supplementary Data

ICMJE author disclosure forms

Video 1 Diagnostic arthroscopy was performed using a standard anterolateral portal for the 45° arthroscope, and the pattern of meniscal tears was evaluated by carefully probing the meniscal tissues. The posteromedial compartment was assessed through the trans-notch view. A 20-gauge spinal needle was inserted into the posteromedial compartment of the knee and used as a probe to assess instability of the meniscocapsular junction. When the meniscocapsular junction was significantly unstable and detached from the posterior edge of the tibial plateau and the posterior horn of the medial meniscus was associated with a meniscal body defect, we used the all-suture anchor technique from an additional posteromedial portal. The tibial posterior margin cartilage was abraded with a shaver to create bleeding from the subchondral bone and to promote healing. Two or 3 all-suture anchors were placed at the tibial margin through a posteromedial portal using a curved anchor guide. The first anchor was set on the medial side of the medial tibial plateau far from the posterior root of the medial meniscus and then toward the lateral side to avoid obstructing the arthroscopic view while working. A 90° angled suture hook device loaded with nitinol wire was inserted through the posteromedial portal and passed through the posterior capsule behind the meniscocapsular junction. One of the suture tails and the wire of the suture hook were retrieved together from the posteromedial portal, and the suture relay was carried out outside the joint. The mattress sutures were tied with a sliding knot using a knot pusher, and the suture tails were cut with a suture cutter. This step was repeated for the remaining suture limbs of each anchor. A cannula was not preferred because it limits mobility; however, if the anchor sutures were difficult to retrieve or handle, a cannula was placed on the posteromedial portal. The anchors had to be placed 1 to 2 mm anterior to the posterior edge of the tibia, and the suture hook had to penetrate slightly posteriorly to the meniscocapsular junction to restore proper tension on the posterior capsule and increase the stability of the femorotibial joint. After all the knots were tied, a probe was used to check the stability of the meniscocapsular junction and posteromedial capsule. Second-look arthroscopy and implant removal surgery were performed between 12 and 24 months after surgery. In all cases in which second-look arthroscopy was possible, the meniscocapsular complex was firmly attached to the tibial plateau.
